# Fast automated yeast cell counting algorithm using bright-field and fluorescence microscopic images

**DOI:** 10.1186/1480-9222-15-13

**Published:** 2013-11-11

**Authors:** Dongpyo Hong, Gwanghee Lee, Neon Cheol Jung, Moongu Jeon

**Affiliations:** 1 Applied Computing Lab., GIST, Oryong-dong, Gwangju 500-712, Korea; 2 Logos Biosystems Inc, Pyungchon-dong, Kyunggi 431-755, Korea

**Keywords:** Fast automated counting, Quantitative measurement, Yeast counting, Dual fluorescence

## Abstract

**Background:**

The faithful determination of the concentration and viability of yeast cells is important for biological research as well as industry. To this end, it is important to develop an automated cell counting algorithm that can provide not only fast but also accurate and precise measurement of yeast cells.

**Results:**

With the proposed method, we measured the precision of yeast cell measurements by using 0%, 25%, 50%, 75% and 100% viability samples. As a result, the actual viability measured with the proposed yeast cell counting algorithm is significantly correlated to the theoretical viability (R^2^ = 0.9991). Furthermore, we evaluated the performance of our algorithm in various computing platforms. The results showed that the proposed algorithm could be feasible to use with low-end computing platforms without loss of its performance.

**Conclusions:**

Our yeast cell counting algorithm can rapidly provide the total number and the viability of yeast cells with exceptional accuracy and precision. Therefore, we believe that our method can become beneficial for a wide variety of academic field and industries such as biotechnology, pharmaceutical and alcohol production.

## Background

Yeast is an invaluable organism in biological research and industry. It is the simplest unicellular eukaryote. Its small genome size and unicellularity make yeast one of the most central model systems in cell biology and genetics, especially when studying cell cycle regulation and signal transduction. Yeast converts carbohydrates to two products, carbon dioxide and alcohol [1]. The former has been utilized for baking and the latter for brewing alcoholic beverages for thousands of years. Recently, the alcohol producing capability of yeast has been applied to bioethanol production using corn and sugar cane [2].

The current method of counting the number of yeast cells is the ASBC (American Society of Brewing Chemists) method [3]. In this protocol, yeast cells are stained with methylene blue to indicate whether they are viable. The methylene blue is unable to penetrate viable cells leaving them unstained. However dead cells are unable to keep the methylene blue from penetrating the cell membrane, staining the cells blue. Thus, dead yeasts are stained in blue while live yeasts are not. Because of the small size, yeast cells need to be observed in high magnification (e.g., 40× or higher objective lens). The magnification is inversely proportional to FOV (Field Of View). Researchers have to count both live and dead yeasts in the small square of the hemocytometer, move the microscope stage to cover a neighboring square and count live and dead yeasts in the next square. Counting continues until a total 0.1 μl counting volume is reached. The central big tile of the hemocytometer corresponds to 25 small squares, and it is a tedious and error-prone process [4]. The statistical significance of the ASBC method is low (25% error is typical) due to human errors, human interpretation and low counting volume (0.1 μl). Moreover, in case of messy cultures (especially occurring in beer and wine brewing and bioethanol production), manual counting is even more challenging. Researchers need to distinguish yeast cells and non-cellular debris (hop in the beer brewing, grape in the wine brewing and corn mash in the bioethanol production).

In order to overcome the difficulties, many automated cell-counting techniques have been proposed by utilizing digital image processing techniques with microscopic images [5-7]. In spite of such developments and their importance, there have been little research efforts on yeast cell counting [8,9]. In this study, we propose a fast automated yeast cell counting algorithm using bright field and fluorescence microscopic images. In addition, we elaborate our investigations on the characteristics of yeast cells for counting them accurately. Finally, we validate our proposed algorithm by evaluating its accuracy, precision, and speed. We describe our proposed algorithm and the results in the following sections.

## Results and discussion

### Assay principle for yeast cells

AO (Acridine orange) and PI (Propidium iodide) are nucleic acid-binding fluorescent dyes. A cell membrane permeable dye, AO can stain nucleic acids of both live and dead cells so that the entire population can be visualized. A cell membrane impermeable dye, PI can only enter and visualize dead cells, of which membrane integrity is compromised. Combining AO/PI dual staining and the automated cell counting system enables the efficient distinction between live and dead cells, as well as the accurate determination of their cell numbers from various origins. However, in case of yeast, the story is a little different. The fluorescence intensities of AO and PI are proportional to the genome size of the cells. Because a yeast cell has 280-times smaller genome than a human cell, the AO fluorescence from the yeast cell is 280-times weaker and the counting based on the weak signal is neither accurate nor precise. To count various cells containing the small amount of genome, an alternative cell staining dye is required.

**Figure 1:**
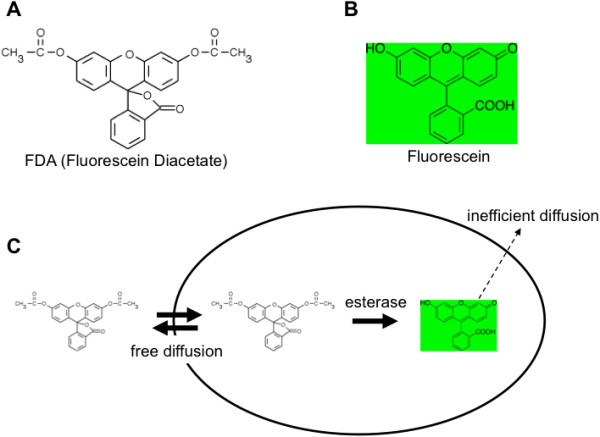
**Working principle of yeast counting by using FDA. (A)** Chemical formula of FDA. **(B)** Chemical formula of Fluorescein. **(C)** Chemical reaction in a yeast cell.

FDA (Fluorescein diacetate) is a fluorogenic cell viability probe. Due to its membrane-permeable nature, FDA can freely move in and out of the plasma membrane of diverse organisms from bacteria to mammalian cells. The internalized FDA is cleaved by intracellular esterases and converted to fluorescein. Fluorescein has an excitation maximum at 494 nm and an emission maximum at 521 nm, so it is compatible with the filter set for the green channel of conventional microscopes. The converted fluorescein carries one negative charge because of carboxylic acid located at the carbon numbered 3, so it cannot cross the plasma membrane anymore. Its retention capability requires an intact plasma membrane. Therefore, FDA staining measures the metabolic activity of esterase enzymes and plasma membrane integrity. The overall working principle is summarized in Figure 1.

### Proposed algorithm

**Figure 2:**
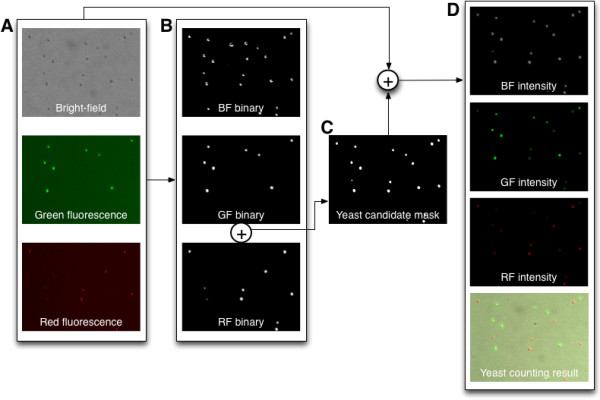
**Overall procedure of the proposed yeast counting algorithm. (A)** The acquired color images (Figure 3A and 3B). **(B)** Binary images from **(A)**. **(C)** Yeast candidate mask image. **(D)** Intensity images by combining **(A)** and **(C)**, and the final counting result image.

Our proposed yeast cell counting algorithm conceptually consists of two main components. The first component is finding objects of interest as yeast candidates. The second component is determining the yeast candidates as yeast cells based on various conditions such as size, intensity level, roundness, and so on. Figure 2 shows the overall procedure of the yeast cell counting algorithm.

**Figure 3:**
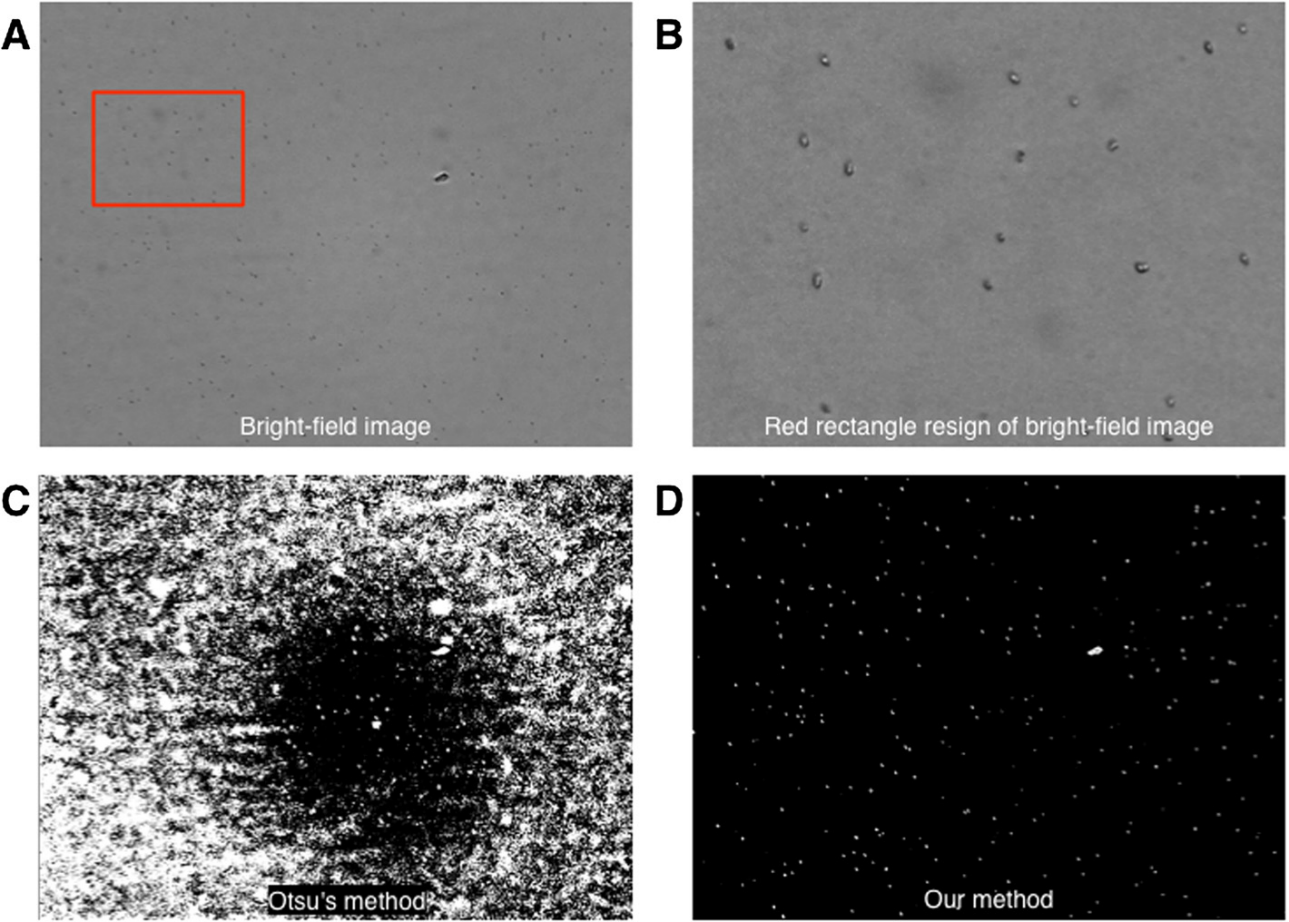
**Bright-field and binary images. (A)** The acquired bright-field images. **(B)** A regional image (640 × 480) of the original **(A)** (2592 × 1944). **(C)** Binary image using Ostu’s method. **(D)** Binary image using our method.

As shown in Figure 2A (from Figure 3A and 3B), the acquired bright-field image is converted to a gray scale image. In addition, green and red fluorescence images are separated by green and red channels, respectively. This channel separation of the acquired color images can reduce memory size as well as counting time because the system deals with 8-bit images instead of 24-bit images. Noise removal and threshold operations are performed on the separated images in order to handle them more efficiently. The binary images also enable a reduction of computing time because the system uses 2-bit rather than 8-bit images. With the binary images (Figure 2B), a yeast candidate mask image can be constructed (Figure 2C) by combining the GF (green fluorescence) and RF (red fluorescence) binary images. By combining the yeast candidate mask image and the acquired original color images (Figure 2D), live and dead yeast cells by comparing the GF intensity and RF intensity values, as well as debris can be discriminated by comparing the gray intensity values in samples. In addition, yeast cell size can be determined by utilizing bright-field image, which enables accurate size calculation. This is in sharp contrast to other methods using only fluorescence microscopic images to measure cell size. It is well known that the cell sizes soly measured by fluorescence images are severly affected by the exposure level of a camera and brightness level of a light source [10,11].

To achieve a best binary image, Otsu’s method has been widely adopted [5,7,12]. In the case of yeast cells, however, we found that Otsu’s method [13] and other similar methods [14] are not applicable if the number of yeast cells is too small or intensities of yeast cells are hardly distinguishable from the background microscopic images as shown in Figure 3C. When we observe the histograms of intensities in the image of yeast samples, they are easily modeled as a Gaussian distribution. From this observation, the threshold value is mapped by function of mean and standard deviation of the image as follows.

$$\mu \;=\;\frac{1}{\mathrm{MN}}\sum_{\chi =0}^{M-1}\sum_{y=0}^{N-1}I\left(\chi ,y\right)$$

$$\sigma \;=\;\sqrt{\frac{1}{\mathrm{MN}}\sum_{\chi =0}^{M-1}\sum_{y=0}^{N-1}{\left(I\left(\chi ,y\right)-\mu \right)}^{2}}$$

T=μ−α⋅σ

where *I(x,y)* is an intensity level of *x* and *y* positions in the given image with *M* width and *N* height; *μ* and *σ* are the mean and standard deviation of the given image, respectively. To estimate the threshold value, *T*, of the given image, we use the confidential interval constant *α*. By using *T*, we can make binary images like Figure 2B and 3D. From our observations in various yeast cell samples, it is sufficient to use the value of *α* as 3.

### Viability comparison

To determine the FDA staining principle which can be applied to yeast counting, we applied FDA/PI mixture to overnight cultured yeast cells.

**Figure 4:**
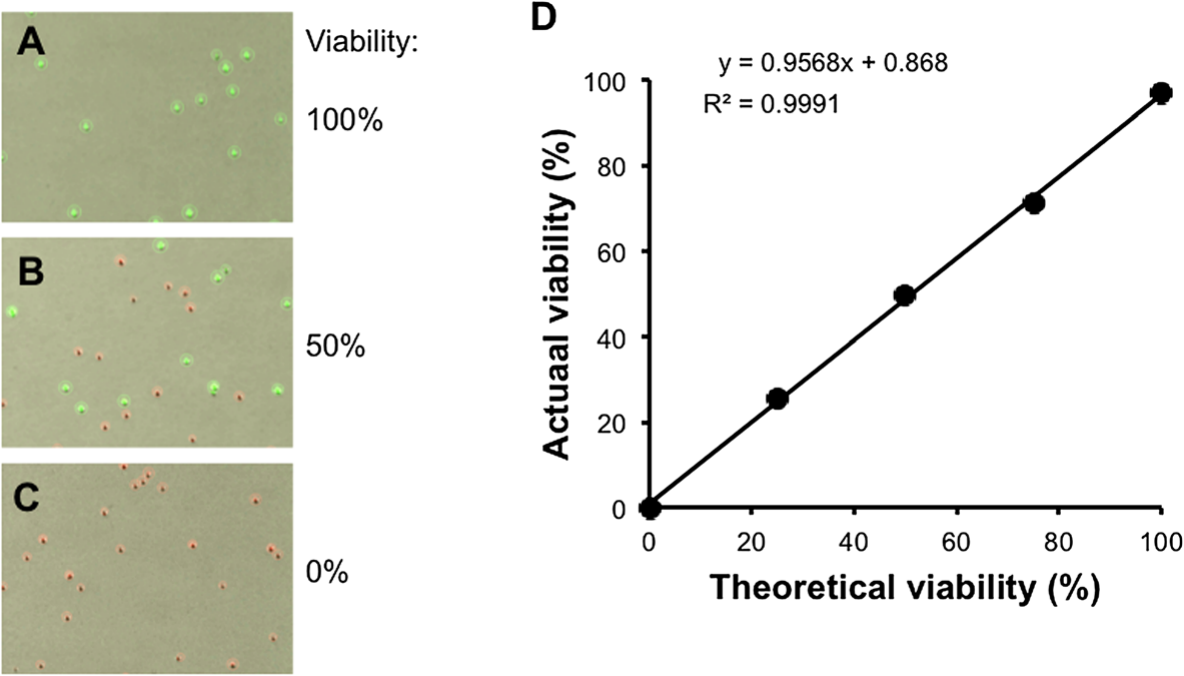
**Counting yeast cells by using the FDA/PI dye. (A)** 100% viability. **(B)** 50% viability. **(C)** 0% viability. **(D)** Correlation of actually counted viability and theoretical viability (R^2^ = 0.9991).

As shown in Figure 4A, FDA can successfully stain all yeast cells when a yeast sample of 100% viability was tested. All yeast cells visible in the bright field image were counted and marked with the green circle. To see if FDA/PI staining can distinguish live and dead yeast cells, we intentionally killed yeast cells by heating at 70°C for 30 minutes (0% viability sample), and a 50% viability sample was prepared by mixing 100% and 0% viability samples at a ratio of 1:1. As seen in Figure 4B and 4C, FDA/PI staining successfully distinguishes live from dead yeasts even when they are mixed in a single vial. Like the 100% viability sample, all yeast cells were counted and labeled with green or red circles according to their health status. We next measured the accuracy of yeast cell viability by using 0%, 25%, 50%, 75% and 100% viability samples. As shown in Figure 4D, the actual viability measured with the proposed yeast cell counting algorithm is significantly correlated to the theoretical viability with the coefficient of correlation (R^2^ = 0.9991). Taken together, the FDA/PI staining method combined with the proposed algorithm can accurately count live and dead yeast cells.

Unlike AO/PI staining, in which two nucleic acid binding dyes bind instantly to genomic DNA, FDA staining makes use of enzymatic reactions for generating fluorescence. Thus, it takes time to reach detectable fluorescence intensity. To measure the optimal time required for fluorescein detection, FDA was added to 100% live yeast cells, and the average fluorescence intensity of objects (yeast cells) was measured at 0, 5, 10, 15, 20 and 30 minutes after the addition of the dye.

**Figure 5:**
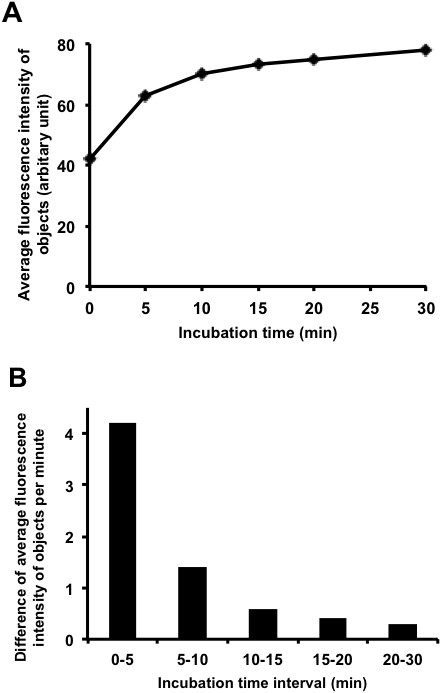
**Kinetics of yeast staining by FDA. (A)** Average fluorescence intensity level of objects according to incubation time. **(B)** Difference of average fluorescence intensity of objects according to incubation time interval.

As shown in Figure 5A, at 0 or upon addition of the dye, the FDA fluorescence is not distinguishable from the background fluorescent intensity level (i.e., around 40 intensity level). Within the first 5 minutes, the yeast cells rapidly gained the fluorescence signal. The rate of the fluorescein accumulation dropped significantly during the next 5 minutes and reached a plateau at 10 minutes after the reaction (Figure 5B). For optimal yeast counting, 10 minutes incubation time of yeast cells with FDA is ideal.

**Figure 6:**
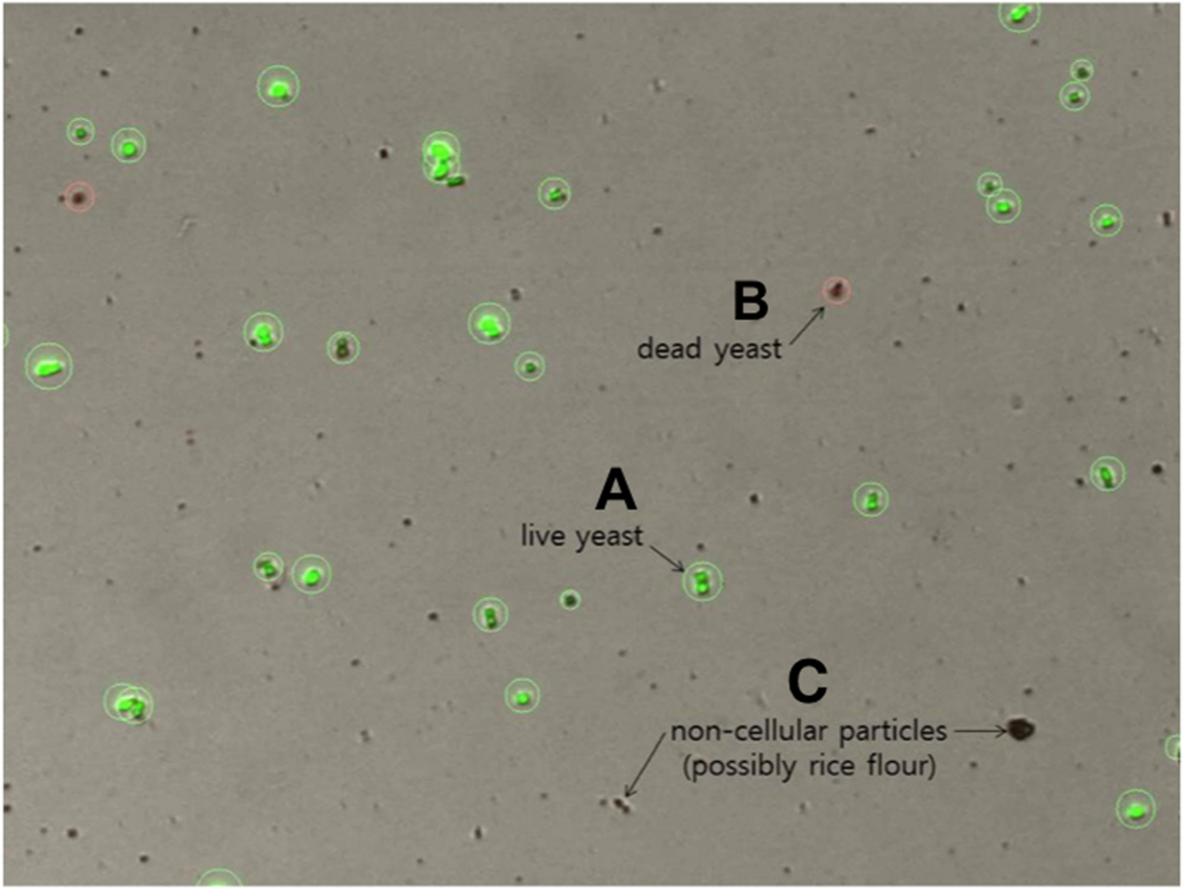
**Yeast counting results in messy culture. (A)** Green circles indicate live yeast cells. **(B)** Red circles indicate dead yeast cells. **(C)** Non-cellular particles or possibly rice flour.

Next, we tested if the proposed algorithm can accurately count the total number and measure the viability even in the presence of non-cellular debris. A traditional Korean rice wine was chosen as the example of a messy culture, which can be readily accessible in local markets. Figure 6 shows that the algorithm can successfully distinguish yeast cells from the non-cellular debris (or possibly rice flour). It is also noteworthy that live yeasts were successfully distinguished from dead yeasts even in the messy culture.

### Counting performance comparison

**Figure 7:**
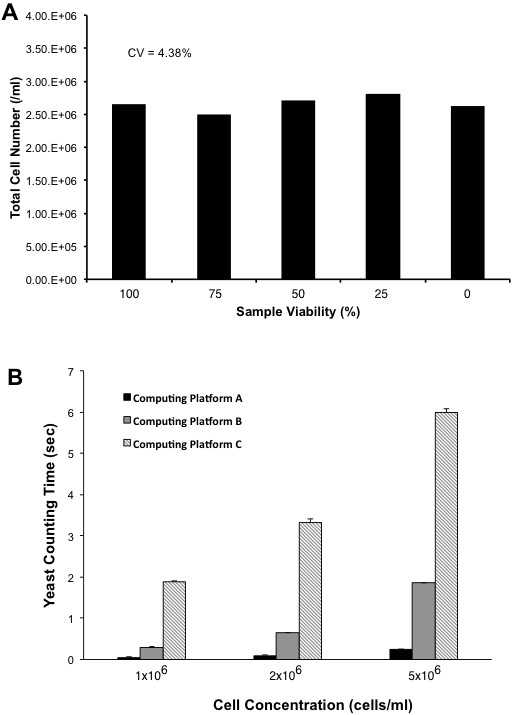
**Counting stability and performance. (A)** Yeast cell viabilities are ranged from 100% to 0%. Average cell numbers are 1,140 cells/ml, and CV is 4.8% that is significantly lower than ABSC method. **(B)** Yeast cell concentrations are ranged from 1 × 10^6^ cells/ml (437 cells in the sample image) to 5 × 10^6^ cells/ml (2,360 cells in the sample image). Average counting time is less than 2 seconds in a conventional PC and less than 6 seconds in an embedded system.

To determine the precision of the proposed algorithm, we counted the concentration of yeast cells with different viabilities from 0% to 100%. Figure 7A shows that the counting results are stable at different viabilities. The CV (coefficient of variation) of counted cells among the different viabilities is 4.38%, which is significantly lower than ASBC method.

To validate the speed of the proposed algorithm, we used different yeast cell concentration samples from 1 × 10^6^ to 5 × 10^6^ cells/ml (i.e., ranging from 437 to 2,360 cells in the sample images, respectively). Furthermore, we also deployed our algorithm into various computing platforms (Table 1) in order to validate feasibility of the proposed algorithm. In this condition, we counted the samples 10 times. Figure 7 shows the result of counting performances on different samples and different computing platforms.

**Table 1:** Computing platforms for counting speed evaluation

	**A**	**B**	**C**
CPU	Intel 3.4GHz	AMD 1.8GHz	Cortex A8 800 MHz
RAM	2 GB	512 MB	512 MB

As shown in Figure 7B, the proposed algorithm counts yeast cells in less than 2 seconds in conventional computing platforms for most cases (e.g., 5 × 10^6^ cells/ml ≈ 2,360 cells in a 2529 × 1944 image size) except for computing platform C. At each trial, the standard deviation of counting time is less than 90 ms. From this experimental result, our algorithm is correlated to CPU clock speed and RAM size. However, it can be negligible if the CPU clock speed and RAM size are at least as powerful as computing platform A. Therefore, the proposed yeast counting algorithm can be applied to any computing platform without loss of its performance.

## Conclusions

The proposed yeast cell counting algorithm can calculate the total number and measure the viability of yeast with exceptional accuracy and precision within less than 2 seconds in a conventional PC and 6 seconds in an embedded system when combined with FDA/PI and yeast dilution buffer. Because our algorithm can count yeast cells in messy culture as well as in pure culture, this algorithm can benefit a wide variety of fields in academics, biotechnology, pharmaceuticals and the alcohol industry.

## Materials and methods

### Methods

*Saccharomyces Cerevisiae* were cultured overnight in the YPD (Yeast Extract Peptone Dextrose) medium. Confluent yeast were diluted at 1:100 and cultured for an additional three hours for mid-log phase cells. After that, cells were again diluted at 1:100 with yeast dilution buffer depending on the concentration of the yeast cells. By using a centrifuge, the supernatant was discarded and the pellet was suspended with the yeast dilution buffer (i.e., the mid-log phase yeast). In order to prepare 0% viability of yeast cells, we heated the yeast sample at 70°C for 30 minutes. Then, we incubated the yeast for 10 minutes at room temperature. For viabilities above 0%, we added 1 μl of FDA and 1 μl of PI to the yeast sample, and incubated it for 10 minutes at room temperature. For the counting, we load 10 μl of the stained yeast sample on the counting slide. Finally, we wait for about 1 minute or until all yeast cells are immobile, then we take images from Luna-FL™ (Logos Biosystems, Korea). To test if the yeast counting algorithm can successfully count the total number of yeasts and calculate the viability in a messy culture, a bottle of traditional Korean rice wine was purchased from a local store. After thorough agitation to make homogenous suspension, the rice wine was diluted at 1:100 yeast dilution buffer. Then, we added 1 μl of FDA and 1 μl of PI to the 18 μl of yeast sample. After the incubation at room temperature for 10 minutes, the yeast staining solution was loaded onto the counting slide of Luna-FL™ and the loaded yeast sample images were acquired from Luna-FL™.

### Images

The samples used for the experiments are FDA/PI-dyed high-resolution images from Luna-FL™. The acquired each sample has 2529 × 1944 image resolution, and also each sample has bright-field, green and red fluorescence images as a set of the experimental data.

### Image processing

The algorithm was implemented with C++ computer language as well as OpenCV (Open Source Computer Vision) library [15]. However, other languages such as *Python* or *Java* can be used to utilize OpenCV library. The following functions were used to implement the proposed algorithm. To separate a color image into green, red, and blue channels, *split* function was used (Figure 2A). A binary image was obtained by *threshold* function (Figure 2B). To make yeast candidates mask image, *bitwise_and* function was applied to GF binary image and RF binary image (Figure 2C). In addition, *bitwise_and* function was used to make intensity images (Figure 2D). To make overlay image, *merge* function was used (Figure 2D). Furthermore, *findContours* function was used to find objects from binary images. Meanwhile, the proposed algorithm is also commercially available with the Luna-FL™ automated cell counter (Logos Biosystems, Korea). The Luna-FL™ automated cell counter automatically processes all the steps shown in Figure 2 including microscopic image capture, image processing, and data display.

## Abbreviations

ASBC: American society of brewing chemists; FOV: Field of view; AO: Acridine orange; PI: Propidium iodide; FDA: Fluorescein diacetate; GF: Green fluorescence; RF: Red fluorescence; DNA: Deoxyribonucleic acid; CV: coefficient of variation; CPU: central processing unit; RAM: random-access memory; YPD: Yeast extract peptone dextrose; OpenCV: Open source computer vision.

## Competing interests

The authors (excluding MJ) declare that they have a competing financial interest in Logos Biosystems, Inc.

## Authors’ contributions

All authors have read and approved the final manuscript. DH implemented yeast counting algorithm, analyzed data, drafted the manuscript, and edited the manuscript. GL carried out yeast counting assay experiments, contributed to conception and design of the study, participated in data analysis and interpretation, and edited the manuscript. NCJ and MJ supervised the algorithm and experiment, contributed to conception and design of the study, and was responsible for final revisions of manuscript.
